# Totally Percutaneous Fetoscopic Repair of Open Spina Bifida: A Single-Center Experience with Perinatal, Obstetric, and Early Functional Outcomes

**DOI:** 10.3390/jcm15114088

**Published:** 2026-05-25

**Authors:** Yuval Gielchinsky, Roie Alter, Ana Idelson, Kinneret Tenenbaum-Gavish, Nir-Ram Duvdevani, Arie Jaffe, Arnon Wiznitzer, Ido Ben Zvi, Ivan Novizki, Eyal Elron, Gil Klinger, Sharon Orbach-Zinger, Denise Araujo Lapa, Amir Kershenovich

**Affiliations:** 1 Fetal Medicine Center, Helen Schneider Hospital for Women, Rabin Medical Center, Petach Tikvah 4941492, Israeltgavishkn@gmail.com (K.T.-G.); jaffea@gmail.com (A.J.);; 2 Gray Faculty of Medical and Health Sciences, Tel Aviv University, Tel Aviv 6997801, Israel; 3 Department of Obstetrics and Gynecology, Hadassah Medical Center, Faculty of Medicine, Hebrew University of Jerusalem, Jerusalem 9112001, Israel; 4 Division of Pediatric Neurosurgery, Schneider Children’s Medical Center of Israel, Petach Tikvah 4920235, Israel; 5 Department of Neonatology, Schneider Children’s Medical Center of Israel, Petach Tikva 4920235, Israel; 6 Department of Anesthesia, Beilinson Hospital, Rabin Medical Center, Petach Tikva 4941492, Israel; 7 Fetal Surgeon at Einstein-Hospital Israelita, São Paulo 05652-900, Brazil

**Keywords:** fetal medicine, fetoscopy, spina bifida, totally percutaneous repair, SAFER technique, intrauterine surgery

## Abstract

**Objectives**: To evaluate perinatal, obstetric, and early functional outcomes following totally percutaneous fetoscopic repair of open spina bifida (OSB) using the skin-over-biocellulose for antenatal fetoscopic repair (SAFER) technique. **Methods**: This retrospective cohort study included all fetuses who underwent totally percutaneous fetoscopic repair of OSB at a single tertiary referral center between September 2018 and March 2026. Eligibility was based on predefined clinical criteria. Data on maternal, fetal, operative, and neonatal outcomes were collected. The primary outcomes included perinatal and obstetric results, need for cerebrospinal fluid (CSF) diversion within the first year of life, and early functional outcomes, including motor improvement and ambulation. Exploratory analyses were performed to assess associations between procedural factors and obstetric outcomes. **Results**: Twenty-two fetuses underwent fetoscopic repair. The procedure was completed in 95% of cases. Median gestational age at surgery was 28.2 weeks. Preterm prelabor rupture of membranes (PPROM) occurred in 68% of cases, and the median gestational age at delivery was 32.7 weeks. All fetuses were live-born, with no neonatal deaths. Maternal outcomes were favorable, with no maternal mortality and no ICU admissions. Vaginal delivery was achieved in 50% of cases, with no uterine rupture or dehiscence. The rate of CSF diversion within the first year was 31.8%. Reversal of hindbrain herniation was observed in 95% of cases. A motor improvement of ≥2 levels was achieved in 68% of patients, with no cases of functional deterioration. Community ambulation at ≥24 months was observed in 53% of cases with available follow-up. **Conclusions**: Totally percutaneous fetoscopic repair of OSB using the SAFER technique was associated with favorable perinatal, obstetric, and early functional outcomes in this single-center cohort. The high incidence of PPROM and subsequent preterm delivery remains an obstetrical challenge. Maternal safety profile, including the preservation of uterine integrity for vaginal delivery, and the early neurological benefits were encouraging. Our results support the feasibility of this approach and highlight the absolute necessity of multidisciplinary expertise and structured training.

## 1. Introduction

Neural tube defects represent a group of congenital anomalies resulting from incomplete closure of the neural tube during early embryogenesis, with open spina bifida (OSB) being among the most common and clinically significant forms [1]. Prenatal diagnosis is typically established through routine obstetric ultrasound, often as early as the first trimester [2,3]. OSB is associated with substantial lifelong morbidity, including motor impairment, neurogenic bladder and bowel dysfunction, and varying degrees of cognitive and neurological sequelae, frequently necessitating complex multidisciplinary care [4]. Traditionally, management of OSB has relied on postnatal surgical repair [5]. However, this approach does not prevent ongoing intrauterine injury to the exposed neural tissue, which may contribute to progressive neurological damage during gestation. This concept led to the development of prenatal surgical repair, which has been shown to improve outcomes compared to postnatal management [6]. Currently, three major intrauterine surgical approaches for OSB repair have been developed. Open fetal surgery, performed via hysterotomy, allows direct multilayer closure of the defect. In an effort to reduce maternal morbidity, minimally invasive alternatives have been introduced [7,8] including a hybrid fetoscopic approach, in which the uterus is exposed via laparotomy prior to insertion of fetoscopic ports, and a totally percutaneous fetoscopic approach, in which laparoscopic ports are inserted percutaneously without laparotomy [9,10,11,12,13]. These approaches differ in terms of surgical access, maternal risk profile, and potential obstetric outcomes. Fetoscopic techniques aim to preserve the benefits of prenatal repair and minimize maternal morbidity, important questions remain regarding their obstetric outcome. In particular, the risks of preterm premature rupture of membranes (PPROM) and preterm delivery, as well as the impact of procedural factors such as gestational age at surgery, operative duration, and technical variation, are still being studied. In addition, the relationship between surgical timing and pregnancy outcomes remains an area of ongoing investigation. Our center implemented the totally percutaneous fetoscopic technique for OSB repair, the skin-over-biocellulose for antenatal fetoscopic repair (SAFER) technique.

This study describes our single-center experience with this approach and evaluates perinatal, obstetric, and early neonatal functional outcomes.

## 2. Materials and Methods

### 2.1. Study Population

A retrospective cohort study was conducted, including all consecutive fetuses who underwent totally percutaneous fetoscopic repair of open spina bifida (OSB) between September 2018 and March 2026 at Rabin Medical Center, a tertiary referral center for fetal medicine and currently the only center in Israel performing prenatal surgical treatment for this condition. All patients referred to our fetal medicine center with a prenatal diagnosis of OSB underwent comprehensive multidisciplinary evaluation by specialists in fetal medicine and pediatric neurosurgery. Following this assessment, detailed counselling was provided to the parents regarding available management options, including expectant management with postnatal repair, termination of pregnancy, and prenatal surgical intervention. The study population comprised patients who elected to continue the pregnancy and undergo prenatal fetoscopic repair.

### 2.2. Inclusion Criteria

Eligibility for prenatal intervention was determined according to predefined clinical criteria. These included: singleton pregnancy, gestational age ≤ 31 weeks at the time of the procedure, and absence of significant additional structural anomalies. All cases were required to have an open spinal lesion with an upper anatomical level between T1 and S1, evidence of hindbrain herniation, and absence of severe kyphosis (defined as fetal kyphosis ≤ 30°), according to previously published SAFER eligibility criteria [14]. In addition, cases with a shortened cervix (<20 mm) were not considered eligible for prenatal repair. All cases underwent genetic evaluation, including chromosomal microarray analysis. Additional exome sequencing was offered when appropriate. In one case, due to advanced gestational age at presentation and patient preference, prenatal genetic evaluation was not performed prior to surgery; postnatal testing revealed an unbalanced translocation involving chromosomes 3 and 10.

### 2.3. Surgical Technique

Patients entered the high-risk pregnancy ward 24 h before their procedure. Preoperative treatment included 100 mg rectal indomethacin and two 12 mg intramuscular doses of betamethasone spaced 24 h apart. All patients underwent percutaneous repair via the Skin-over-Biocellulose for Antenatal Fetoscopic Repair (SAFER) technique [13,15]. The procedure was performed under total intravenous anesthesia (TIVA) [16]. Anesthesia induction was performed using propofol (1.5 mg/kg), rocuronium (1 mg/kg), and fentanyl (150–250 μg). Maintenance of anesthesia was achieved using continuous propofol infusion (100–200 μg/kg/min) and remifentanil infusion (0.05–0.2 μg/kg/min). A bispectral index (BIS) monitor was used in all patients to guide depth of anesthesia.

An arterial line was inserted for continuous hemodynamic monitoring and rapid management of potential intraoperative maternal instability. Prior to incision, 2 g of cefazolin was administered intravenously as a prophylactic agent. Under ultrasound guidance, an 18-gauge percutaneous needle was used to perform amnioinfusion with 500 mL of warmed Ringer’s lactate solution. Using the Seldinger technique, four trocars were inserted into the uterus: one 5-mm balloon-tipped laparoscopic trocar (Applied Medical, Rancho, Santa Margarita, CA, USA) and three 11-Fr introducer (Terumo, Tokyo, Japan) sheaths. Amniotic fluid was aspirated and preserved in a sterile container at 37 °C. The uterine cavity was then insufflated with heated, humidified CO_2_ (Lexion Medical, St Paul, MN, USA). Intrauterine pressure was maintained at 3–5 mmHg above baseline uterine pressure (mean 14 mmHg, range 10–18 mmHg), with a gas flow limit of 30 L/min. The fetus was positioned using standard 3-mm laparoscopic instruments (Karl Storz (Tuttlingen, Germany), ConMed (Largo, FL, USA), AescoLap (Tuttlingen, Germany)) under fetoscopic visualization. The neural placode was circumferentially dissected and released at the transition zone, and the skin was further undermined to allow proximation of the edges in the midline. The placode was covered with a biocellulose patch (Bionext, Rana Paraná, Brazil). When primary skin approximation was feasible, the skin was closed over the patch with 2-0 monofilament suture (non-absorbable propylene) using a single running barbed stitch (Quil, SRS, Angiotech, Reading, PA, USA). When possible, a myofascial flap was created and approximated in the midline above the a biocellulose patch using absorbable barbed suture (2-0 synthetic absorbable PDO, Quil). In cases of larger defect that was too large to allow skin approximation, two different patches were used: a bi-laminar skin substitute (Nevelia, Symatese, Chaponost, France) placed on top of the biocellulose patch. The skin substitute was attached to the surrounding skin with two running barbed sutures. When possible, a myofascial flap was created and approximated in the midline above a biocellulose patch using absorbable barbed suture [13]. In cases of intraoperative uterine contractions, a bolus dose of 6.75 mg intravenous atosiban was administered, followed by a 300 μg/min maintenance infusion for 3 h, and a subsequent 100 μg/min continuous infusion for 24 h. At the end of the procedure, intrauterine CO_2_ was evacuated, and the preserved amniotic fluid was reinfused, followed by intraamniotic infusion of 2 g cefazolin prior to trocar removal. Postoperatively, 50 mg of oral indomethacin was administered every 12 h for the first 24 h as a tocolytic agent. Patients were discharged after the procedure following an uneventful recovery and with reassuring fetal status.

### 2.4. Follow-Up

Following discharge, patients were evaluated weekly in an outpatient setting. Follow-up included assessment of fetal growth, amniotic fluid volume, cervical length, ventricular size, posterior fossa anatomy, and fetal motor function. In cases of preterm prelabor rupture of membranes (PPROM), patients were managed as inpatients.

### 2.5. Delivery and Neonatal Management

Timing and mode of delivery were determined based on standard obstetric indications, with vaginal delivery permitted. Following delivery, a pediatric neurosurgeon inspected the repair site, and the suture ends were trimmed flush with the skin. In cases involving a bilaminar patch, the material was left in place and protected with a dressing (Mepilex, Gothenburg, Sweden) until complete secondary epithelialization was achieved.

Neonates were admitted to the NICU and were monitored daily for cerebrospinal fluid (CSF) leakage at the repair site, defined as visible leakage or persistent wet dressing. Neurological motor level was determined based on standardized clinical neurological examination performed during routine follow-up by pediatric neurosurgeons experienced in the management of spina bifida patients.

### 2.6. Data Collection

Data were collected from electronic medical records and included maternal, fetal, operative, and neonatal variables. Maternal data included age, body mass index, obstetric history (gravidity and parity), prior uterine surgery, placental location, and cervical length. Fetal characteristics included lesion type (myelomeningocele or rachischisis), upper anatomical level of the lesion, hindbrain herniation, ventricular size, talipes, kyphosis, and fetal biometry. Operative variables included gestational age at surgery, type of anesthesia, number of patches used, use of myofascial flaps and relaxing incisions, gas insufflation, and total operative (skin-to-skin) times, and procedural completion. Maternal and obstetric outcomes included intraoperative and postoperative complications (e.g., gas embolism, maternal blood transfusion, and pulmonary edema), PPROM, chorioamnionitis, duration of postoperative hospitalization, intrauterine fetal demise, delivery mode, and uterine dehiscence. Neonatal outcomes included sex, Apgar scores, birth weight, intraventricular hemorrhage, neonatal sepsis, reversal of hindbrain herniation, motor level at birth, survival, and length of NICU stay. Data regarding the requirement for cerebrospinal fluid diversion (shunt placement) and ambulation status were obtained from the most recent neurosurgical follow-up.

Detailed neurosurgical outcomes, including CSF leakage, wound dehiscence, and reoperations, and the long-term neurological follow-up, are being analyzed separately and will be reported separately.

### 2.7. Statistical Analysis

Statistical analyses were performed using IBM SPSS Statistics for Windows, Version 25.0 (IBM Corp., Armonk, NY, USA). Continuous variables are presented as mean ± standard deviation or median (interquartile range), according to data distribution. Categorical variables are presented as counts and percentages. Exploratory analyses were conducted to evaluate associations between selected clinical and operative variables and outcomes. Comparisons between groups were performed using the Mann–Whitney U test for continuous variables and Fisher’s exact test for categorical variables. Associations between gestational age at surgery and obstetric outcomes were evaluated using Spearman’s rank correlation coefficient (*r_s_*).

Given the limited sample size, subgroup analyses were considered exploratory, and no formal adjustment for multiple comparisons was performed. A two-sided *p*-value < 0.05 was considered statistically significant.

### 2.8. Ethics

The study was approved on 17/4/2025 by the Institutional Review Board of Rabin Medical Center (Approval No. RMC-19-0295), which waived the requirement for written informed consent due to the retrospective nature of the study.

## 3. Results

**Figure 1:**
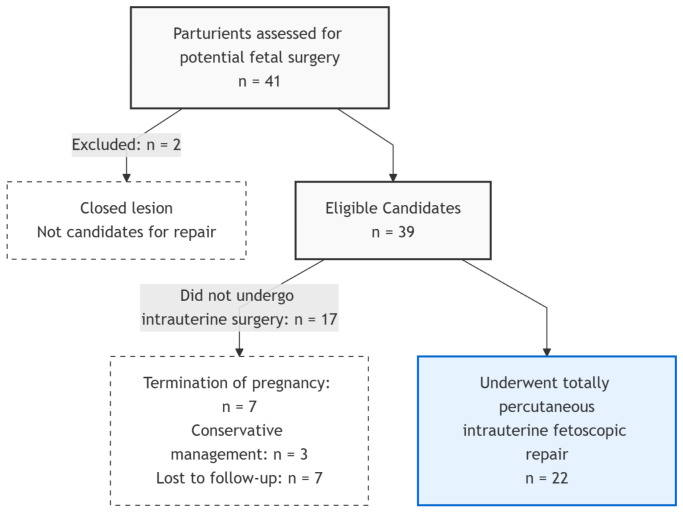
**Flow diagram of patient selection and study cohort.** The chart illustrates the multidisciplinary assessment, exclusions, and alternative management decisions for 41 referred parturients, resulting in a final cohort of 22 patients who underwent totally percutaneous intrauterine fetoscopic repair of open spina bifida. Solid lines indicate eligible and continuing cohorts, while dashed lines represent excluded patients or those who did not undergo the primary surgery. The blue-shaded box highlights the final active intervention group (N = 22).

**Figure 2:**
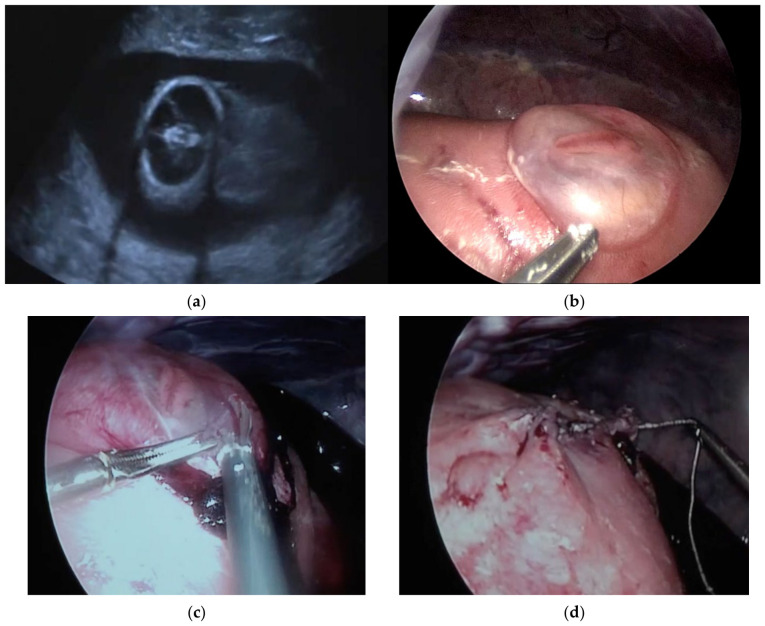
Intrauterine fetoscopic repair of open spina bifida using the SAFER technique: (**a**) Prenatal ultrasound demonstrating the myelomeningocele sac; (**b**) Fetoscopic view of the exposed myelomeningocele sac with identification of the neural placode; (**c**) Dissection and incision of the transitional zone, allowing release and mobilization of the neural placode; (**d**) Final closure demonstrating a watertight repair.

During the study period between 14 December 2018, and 15 December 2025, a total of 41 parturients carrying fetuses with suspected neural tube defects were referred for potential fetal surgery at our center. Following the initial multidisciplinary evaluation, 2 patients were excluded as they were diagnosed with closed lesions, which are not candidates for intrauterine repair. Of the remaining 39 candidates, 7 (17.9%) elected for termination of pregnancy, 3 (7.7%) opted for conservative management with planned postnatal follow-up and intervention, and 7 (17.9%) were lost to follow-up after their initial assessment (Figure 1). The remaining 22 patients met all inclusion criteria and proceeded with totally percutaneous intrauterine fetoscopic repair of open spina bifida using the SAFER technique (Figure 2).

Baseline maternal and fetal characteristics are summarized in Table 1. All cases were singleton pregnancies evaluated in a tertiary referral center and selected for prenatal intervention following multidisciplinary assessment and predefined eligibility criteria. Lesion type was classified as myelomeningocele in 15 cases (68.2%) and rachischisis in 7 cases (31.8%).

### 3.1. Perioperative and Maternal Outcomes

Perioperative and maternal outcomes are summarized in Table 2.

The median gestational age at surgery was 28.2 weeks (IQR 27.9–29.0), with a median operative duration of 220 min (IQR 196–257) and insufflation time of 135 min (IQR 124–162). General anesthesia using total intravenous anesthesia (TIVA) was employed in the majority of cases (95.5%). The procedure was successfully completed in 21 cases (95.5%). One case of gas embolism occurred; the insufflation was immediately discontinued, leading to rapid maternal stabilization, and the procedure was continued in a limited fashion. However, definitive closure was not completed to minimize operative time. Intraoperative maternal complications were uncommon except for one case (4.5%) of gas embolism. Maternal blood transfusion was required in three cases (13.6%), including one case associated with uterine atony under gas anesthesia, after which the protocol was modified to TIVA, and two cases in which transfusion was administered prophylactically due to pre-existing maternal anemia. No cases of pulmonary edema or ICU admission were observed. Six patients (27.3%) remained hospitalized until delivery, primarily due to patient preference, preterm prelabor rupture of membranes, or progression toward delivery. Among patients discharged following the procedure (72.7%), the median postoperative hospital stay was 3.0 days (IQR 2.8–4.3).

Postoperative chorioamnionitis occurred in 2 cases (8.7%). Microbiological findings included *Bacillus cereus* and *Corynebacterium jeikeium*, both identified within the first week following surgery.

Urinary tract infections were identified in six cases (27.3%), all of which were asymptomatic and detected on routine surveillance urine cultures. Identified pathogens included *Escherichia coli* (including ESBL-producing strains), *Pseudomonas* species, and *Streptococcus anginosus*. No cases of intrauterine fetal demise were recorded. PPROM occurred in 15 cases (68.2%; 95% CI: 45.1–86.1%), at a median gestational age of 30.6 weeks (IQR 29.7–32.7), with a median interval of 2.4 weeks (IQR 1.3–4.6) from surgery to membrane rupture. All pregnancies resulted in live births. Delivery occurred at a median gestational age of 32.7 weeks (IQR 30–34.9), with a median latency of 3.7 weeks (IQR 1.7–7.1) from surgery to delivery. Vaginal delivery was achieved in 50.0% of cases, while 11 patients (50.0%) underwent cesarean delivery for standard obstetric indications. No cases of uterine rupture or dehiscence were observed.

### 3.2. Neonatal and Early Functional Outcomes

Neonatal and Early Functional Outcomes are summarized in Table 3.

All fetuses were live-born, with no events of neonatal deaths. One infant died after the neonatal period (day 55 of life) due to ESBL-producing *E. coli* sepsis, which was considered a nosocomial infection acquired during the NICU stay. The median 5-min Apgar score was 10 (IQR 9–10). No intraventricular hemorrhage grade > 2 was observed. The median length of NICU stay was 27 days (IQR 11.75–60.5). Cerebrospinal fluid (CSF) diversion (ventriculoperitoneal shunt) within the first year of life was required in 7/22 infants (31.8%). Reversal of hindbrain herniation was documented in 20/21 infants (95.2%) with available prenatal or postnatal imaging data by neurosonography or brain MRI.

**Figure 3:**
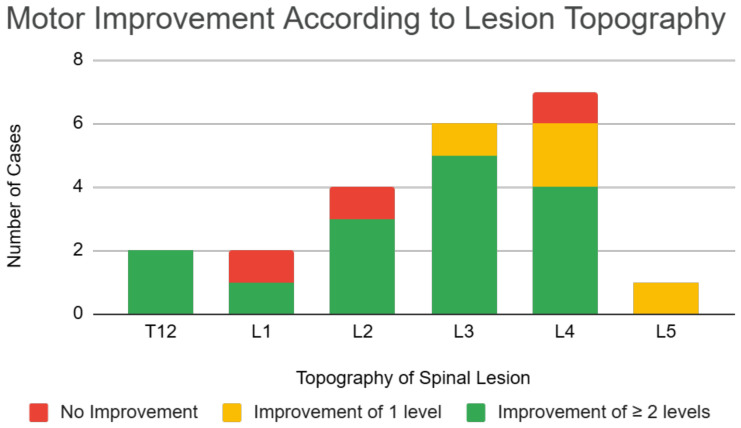
**Motor improvement according to anatomical lesion level.** Distribution of motor improvement stratified by upper anatomical level of the spinal lesion. Most patients demonstrated improvement of ≥2 motor levels across lesion topographies, with no cases of neurological deterioration observed. T12: thoracic level 12; L1–L5: lumbar levels 1 to 5.

Motor function improved relative to the anatomical level of the lesion in the majority of cases: A motor improvement of ≥2 levels was observed in 68.2% of cases, while 18.2% demonstrated a one-level improvement. Three cases (13.6%) showed no improvement, and no cases of neurological deterioration were observed. Exploratory visualization of motor improvement according to lesion topography is presented in Figure 3, showing that motor improvement was observed across different lesions including high levels.

Ambulation data were available for 15 children who reached at least 24 months of age and had adequate follow-up. Of these, 8/15 (53.3%) were community ambulators. One infant died, two were lost to follow-up (back to their home country), and four had not yet reached the age of ambulation at the time of analysis. One infant who was later diagnosed with an unbalanced chromosomal translocation was included in the analysis; exclusion of this case did not materially affect the ambulation results.

### 3.3. Subsequent Pregnancies

Data on subsequent pregnancies were available for six women. Of these, five had uncomplicated vaginal deliveries, while the sixth pregnancy is currently ongoing and in the third trimester. No cases of uterine rupture were reported.

### 3.4. Exploratory Analyses

Exploratory analyses were performed to assess the association between gestational age at surgery and the risk of PPROM. The gestational age at surgery was slightly lower in pregnancies complicated by PPROM compared to those without PPROM (median 28.1 vs. 29.0 weeks), representing a non-significant trend (*p* = 0.12).

To account for differences in time-at-risk, an exploratory analysis was performed where early PPROM (defined as membrane rupture within 14 days of surgery) was analyzed separately and occurred in 7/22 (31.8%; 95% CI: 13.9–54.9%) of cases. This exploratory analysis showed that there was no significant difference in the gestational age at surgery between those who experienced early PPROM and those who did not (*p* = 0.67). Similarly, among cases complicated by PPROM, no significant correlation was observed between gestational age at surgery and latency to membrane rupture (*r_s_* = −0.23; 95% CI: −0.66 to 0.32; *p* = 0.41). No significant correlation was observed in this exploratory analysis between gestational age at surgery and gestational age at delivery (*r_s_* = 0.07; 95% CI: −0.36 to 0.48; *p* = 0.75), or between gestational age at surgery and the surgery-to-delivery interval (*r_s_* = −0.20; 95% CI: −0.57 to 0.24; *p* = 0.35).

## 4. Discussion

In this single-center cohort, totally percutaneous fetoscopic repair of OSB using the SAFER technique was associated with favorable perinatal and early functional outcomes. This work represents our initial experience following structured training and implementation of the technique [12]. The technical success rate of 95% is consistent with previously reported SAFER series for a group within its learning curve [13]. Furthermore, the median operative duration (220 min) fell within the range described in the literature [13,17]. Maternal safety was reassuring: with no maternal mortality, no need for ICU admission, and rapid postoperative recovery, with the majority of patients discharged within a few days. Infectious complications were rare, although two cases of chorioamnionitis were observed, involving pathogens not covered by standard prophylaxis, highlighting the need to further evaluate optimal perioperative antibiotic regimens. From an obstetric perspective, no cases of intrauterine fetal demise were observed, aligning with previous reports using the SAFER technique [13,17].

A limitation of the totally percutaneous technique is the high rate of PPROM, which represents an obstetric challenge. In our cohort, PPROM occurred in 68.2% of cases. While early adopters of this technique initially reported PPROM rates as high as 80% [13,17,18], and recent procedural modifications have reduced this incidence to 56% [15], our rate remains substantially higher than those typically reported for hybrid fetoscopy (32%) and open hysterotomy (31%) [18]. We expect that increased procedural experience, minimizing the need for amnioinfusion, and utilizing fewer trocar ports (e.g., three versus four) may mitigate uterine trauma and help reduce PPROM rates in future cohorts. The mean gestational age at delivery in our cohort (32.7 weeks) was comparable to other fetoscopic series [13,14,15,17], and lower than the reported following open fetal repair [6]. Such indirect comparisons must be interpreted with strict caution due to inherent differences in study design, patient selection, and surgical approaches.

Importantly, half of the patients were delivered vaginally, with no cases of uterine rupture or dehiscence. This represents a major advantage of the fetoscopic approach compared to open fetal surgery, where cesarean delivery is mandatory and uterine scar complications are relatively common [6], leading to fetal death in 40% of the cases [19]. Neonatal outcomes were favorable, with all fetuses live-born and no early neonatal deaths, and were in line with previously reported perinatal mortality rates following open fetal repair and postnatal management [6,18].

The rate of CSF diversion (VP shunt) within the first year of life in our cohort was 31.8%. This finding is consistent with previously reported prenatal repair outcomes. Reported rates of CSF diversion range between 17–38% following open fetal repair, 36–46% after fetoscopic repair, and up to 81% after postnatal repair [18]. Reversal of hindbrain herniation was observed in 95.2% of cases, slightly higher than the pooled data from SAFER cohorts [14], and appears higher than rates reported following postnatal repair [6]. Notably, the majority of patients demonstrated clinical improvement in motor function, with no cases of functional deterioration. A motor improvement of ≥2 levels was observed in 68.2% of cases, appearing favorable when compared with previously reported open fetal surgery outcomes [6].

Community ambulation after 24 months was achieved in 53.3% of patients in our cohort. In the MOMS trial, independent ambulation was reported in 42% of children who underwent prenatal open repair, compared with 21% in those following postnatal repair [6]. Although direct comparisons are limited by differences in outcome definitions and follow-up duration, the ambulation rates observed in our cohort appeared consistent with those reported following prenatal repair, both in open and fetoscopic series [6,18]. The distribution of lesion levels in our cohort was predominantly within the mid-lumbar range, with a smaller proportion of higher lesions, suggesting that the observed outcomes are not limited to low-level defects alone. Long-term urological outcomes were not evaluated in the present study and warrant dedicated future investigation.

Although data on subsequent pregnancies were limited, the observed outcomes were reassuring. All completed subsequent pregnancies resulted in uncomplicated vaginal deliveries with no instances of uterine rupture. Although limited by small numbers, these findings highlight the potential maternal advantages of avoiding hysterotomy, particularly for patients planning future pregnancies or larger family size.

The learning curve associated with fetoscopic repair is significant, with previous studies suggesting that competency requires more than 50 cases [20]. Our findings demonstrated that favorable outcomes can be achieved even in smaller series when the procedure is performed in experienced multidisciplinary settings. Structured training, mentorship, and ongoing collaboration between our center and Dr Lapa’s center at Einstein Hospital, Israelita (Sao Paulo, Brazil) likely play an important role in optimizing our outcome.

The timing of surgery in our cohort was relatively advanced compared to the original MOMS criteria, largely influenced by clinical and logistical factors, including late referrals and the time required to complete genetic evaluations, which constituted part of the institutional eligibility criteria for prenatal repair. Regarding functional outcomes, our findings challenge the traditional paradigm that earlier intervention is invariably superior. While our data, together with recent literature [4], suggest that fetoscopic repair beyond 26 weeks of can be an effective approach, we emphasize that these observations remain purely speculative and require prospective validation. Some newer series have shown a decreased need for spinal cord untethering at school age; while this may be partly attributed to the use of biocellulose patches [21], it is also hypothesized that operating at a later stage alters fetal healing and anatomical repair. However, any potential advantages of later surgery remain hypothesis-generating.

**Figure 4:**
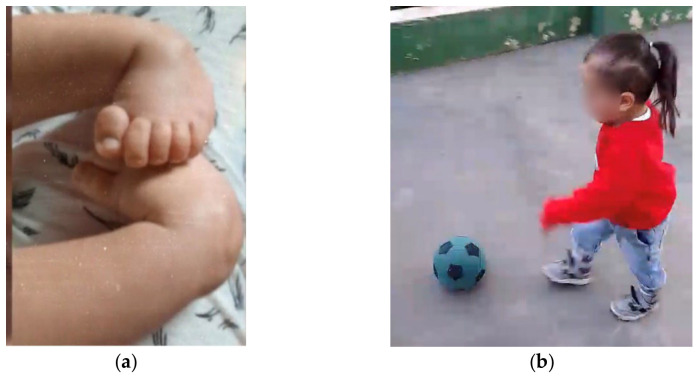
Functional outcome in a child with prenatal clubfoot: (**a**) Prenatal diagnosis of clubfoot by ultrasound, confirmed at birth; (**b**) At 3 years of age, the same child demonstrates independent ambulation and age-appropriate motor function, including running and playing.

Finally, the presence of clubfoot, often considered a marker of more severe neurological involvement, did not uniformly predict poor outcomes in our cohort. Although previous studies have suggested a trend toward worse motor outcomes in such cases [22], and some even consider this condition as an exclusion criteria for prenatal repair [23], our experience indicates that functional outcomes may still be favorable. Therefore, clubfoot should not be considered an absolute contraindication to fetoscopic repair, and exclusion based solely on this finding may be overly restrictive. This observation is further illustrated by a representative case from our cohort (Figure 4), in which a child diagnosed prenatally with clubfoot demonstrated excellent functional outcome at follow-up.

The findings of this study must be interpreted in light of several important limitations. First, this represents a retrospective, single-center early experience with a relatively modest cohort size and no control group. Consequently, our findings are inherently descriptive, which limits both the statistical power of our subgroup analyses and the broader generalizability of the results. Second, the follow-up duration for functional outcomes was limited for some patients, particularly concerning long-term ambulation and late neurological development. Furthermore, early functional assessments were based on standard clinical neurological examinations rather than formal, validated functional scales, and the evaluators were not independent of the treating surgical team. Finally, although exploratory analyses were performed to evaluate potential associations between procedural factors and obstetric outcomes, our small sample size precluded adjustment for multiple comparisons; thus, these statistical findings must be considered strictly hypothesis-generating rather than definitive. Our exploratory analyses regarding the timing of surgery and the subsequent risk of PPROM are inherently limited by time-at-risk bias. Fetuses undergoing totally percutaneous repair at an earlier gestational age naturally have a prolonged exposure window to experience PPROM. Derived metrics such as the surgery-to-PPROM and surgery-to-delivery intervals are structurally correlated with the gestational age at intervention, which can obscure true biological relationships. Future studies must utilize time-to-event frameworks, modeling gestational age at delivery as the primary outcome, to accurately delineate the impact of surgical timing.

## 5. Conclusions

Totally percutaneous fetoscopic repair of open spina bifida using the SAFER technique was associated with favorable perinatal, obstetric, and early functional outcomes in this single-center cohort. The procedure demonstrated a high technical success rate, a reassuring maternal safety profile, and consistent early neurological benefits, including low rates of CSF diversion and overall favorable motor improvement. Importantly, the ability to preserve uterine integrity, allow for future vaginal deliveries, and support uncomplicated subsequent pregnancies, highlights a profound maternal advantage of this fetoscopic technique.

## Figures and Tables

**Table 1:** Baseline maternal and fetal characteristics (N = 22).

Maternal Characteristics	
Maternal age (years)	30.0 ± 6.6
Body mass index (kg/m^2^)	24.8 ± 3.0
Gravidity	4.0 (2–5)
Parity	2 (1–4)
Prior uterine surgery	3 (14%)
Pregnancy characteristics	
Placenta location	
Anterior	10 (45%)
Posterior	10 (45%)
Fundal	1 (5%)
Lateral	1 (5%)
Cervical length (mm)	33.0 (31.3–34.8)
Fetal characteristics	
Lesion type	
Rachischisis (flat lesion)	7 (31.8%)
Myelomeningocele	15 (68.2%)
Upper lesion level	
Thoracic	2 (9.1%)
L1-L2	6 (27.3%)
L3-L4	13 (59.1%)
L5-S1	1 (4.5%)
Ventricular size (mm)	11.5 (10–13)
Hindbrain herniation	22 (100%)
Talipes	2 (9%)
Kyphosis	3 (14%)
Estimated fetal weight (grams)	1035 (906–1140)
Estimated fetal weight percentile	32.5 (18.3–43.8)
Normal genetic evaluation	21 (95%)

Values presented as mean ± SD, median (IQR), or *n* (%), as appropriate.

**Table 2:** Perioperative, maternal, and pregnancy outcomes.

Perioperative Characteristics	
Gestational age at surgery (weeks)	28.2 (27.9–29.0)
Operation duration (skin-to-skin, min)	220 (196–257)
Insufflation time (min)	135 (124–162)
Anesthesia type	
TIVA	21 (95%)
Gas anesthesia	1 (5%)
Number of patches	
One patch	18 (82%)
Two patches	4 (18%)
Use of a muscle flap	8 (36%)
Relaxing incisions	0
Surgery completed	21 (95%)
Intraoperative maternal outcomes	
Maternal ICU admission	0
Gas embolism	1 (5%)
Maternal blood transfusion (intraoperative)	3 (14%)
Pulmonary edema	0
Postoperative maternal outcomes	
Discharged after procedure	16 (72.7%)
Remained hospitalized until delivery	6 (27.3%)
Maternal hospital stay (days) ^$^	3.0 (2.8–4.3)
Chorioamnionitis	2 (8.7%)
Urinary tract infection ^$$^	6 (27.3%)
Pregnancy outcomes	
IUFD	0
PPROM	15 (68.2%)
Gestational age at PPROM (weeks)	30.6 (29.7–32.7)
Latency surgery to PPROM (weeks)	2.4 (1.3–4.6)
Delivery outcomes	
Live birth rate	22 (100%)
Gestational age at delivery (weeks)	32.7 (30.0–34.9)
Surgery-to-delivery (weeks)	3.8 (1.7–7.1)
Vaginal delivery	11 (50%)
Cesarean delivery	11 (50%)
Uterine rupture/dehiscence	0

Data presented as median (IQR) or *n* (%). ^$^ excluding patients that was admitted until delivery, ^$$^ asymptomatic, detected on routine cultures. TIVA, Total Intravenous Anesthesia; ICU, Intensive Care Unit; IUFD, Intrauterine Fetal Demise; PPROM, Preterm Prelabor Rupture of Membranes.

**Table 3:** Neonatal and Early Functional Outcomes.

Birth Weight, g (Mean ± SD)	1714 (1422–2338)
Apgar score at 5 min (median, IQR)	10 (9–10)
Male sex, *n*/N (%)	18/22 (81.8%)
Neonatal death (<28 days), *n*/N (%)	0/22
Post-neonatal death # (>28 days), *n*/N (%)	1/22 (4.5%)
IVH > grade 2, *n*/N (%)	0/22 (0%)
Neonatal sepsis, *n*/N (%)	1/22 (4.5%)
NICU stay, days (median, IQR)	27 (11.8–60.5)
CSF diversion ≤ 1 year (VP shunt), *n*/N (%)	7/22 (31.8%)
Reversal of hindbrain herniation, *n*/N (%)	20/21 (95.2%) *
Motor improvement ≥ 2 levels, *n*/N (%)	15/22 (68.2%)
Motor improvement 1 level, *n*/N (%)	4/22 (18.2%)
No improvement, *n*/N (%)	3/22 (13.6%)
Motor Deterioration, *n*/N (%)	0/22
Community ambulation ≥ 24 months, *n*/N (%)	8/15 (53.3%) **

Data presented as median (IQR) or *n* (%). # Death at age of 55 days due to ESBL *E. coli* sepsis (NICU-acquired); IVH, Intraventricular Hemorrhage; NICU, Neonatal Intensive Care Unit; CSF, Cerebrospinal Fluid. * One missing due to loss of follow-up. ** Four infants were too young for assessment (<24 months), two were lost to follow-up, and one died in the NICU.

## Data Availability

The raw data supporting the conclusions of this article will be made available by the authors on request.

## Abbreviations

The following abbreviations are used in this manuscript:

OSB	Open Spina Bifida
SAFER	Skin-over-biocellulose for Antenatal Fetoscopic Repair
CSF	Cerebrospinal fluid
PPROM	Preterm prelabor rupture of membranes
ICU	Intensive Care Unit
TIVA	Total intravenous anesthesia
CO_2_	Carbon dioxide
NICU	Neonatal Intensive Care Unit
ESBL	Extended-spectrum beta-lactamase
MRI	Magnetic Resonance Imaging
VP	Ventriculoperitoneal
MOMS	Management of Myelomeningocele Study
